# TERT mRNA peaks during meiotic prophase in *Tetrahymena thermophila* as determined by RT–qPCR

**DOI:** 10.17912/micropub.biology.002307

**Published:** 2026-07-26

**Authors:** Lily M. Avila, Dawid J. Oleksy, Shuntaro Nakamura, Gwendolyn S. Geissler, Victoria C. Kutkovska, Karen E. Kirk

**Affiliations:** 1 Biology, Lake Forest College, Lake Forest, IL, United States

## Abstract

Telomerase reverse transcriptase (TERT) expression increases during sexual development in *Tetrahymena thermophila* , when thousands of new chromosome ends require telomere addition. To define the timing of TERT mRNA accumulation, we combined cytological staging with RT–qPCR across conjugation stages. DAPI microscopy was used to score micronuclear and macronuclear morphology. TERT mRNA increased early in conjugation, peaked during meiotic prophase (crescent stage), remained elevated through post-crescent meiosis, and declined during macronuclear development before extensive de novo telomere addition. These data agree with earlier work but provide improved quantitative, temporal and cytological resolution, showing that peak TERT mRNA accumulation precedes large-scale telomere addition.

**Figure 1. Cytological staging and RT–qPCR analysis of TERT expression during conjugation in Tetrahymena thermophila:** **(A)** Percentage of mating pairs per biological replicate and time point classified into cytological stages by DAPI epifluorescence. Time points at 4 h, 5 h, and 9.5 h were selected for further analysis. From these time points, pairs were categorized that had morphologies consistent with the crescent stage, the post-crescent meiosis stage, and the macronuclear development stage, and a small number had an initiation stage similar to starved cells at mixing. **(B)** Representative confocal DAPI-stained images show nuclear morphologies of paired cells at time points enriched for crescent stage, post-crescent meiosis, and developing macronuclei after mixing. Phase-contrast and grayscale DAPI images are shown. Arrows are shown in only one cell per pair: at 4 h, a red arrow indicates the MAC and a green arrow indicates the crescent MIC; at 5 h, products of micronuclear meiosis are indicated with a green arrow (and a red arrow indicates the MAC); and at 9.5 h red arrows indicate the developing MACs, a thin red arrow indicates the old MAC, and a green arrow indicates post-zygotic MICs. **(C)** Relative TERT mRNA expression measured by RT-qPCR during conjugation. Expression peaked during the crescent and post-crescent meiosis stages and remained elevated during macronuclear development. Transcript levels were normalized to Actin and GAPDH and expressed relative to starved cells (1-fold). Bars represent the mean ± SE of three technical replicates from each of three biological replicates. One-way ANOVA indicated statistical significance (p < 0.001). Tukey's post hoc test showed that expression at 4 h, 5 h, and 9.5 h was significantly greater than at 0 h (** and ***), whereas 4 h and 5 h did not differ significantly (ns).

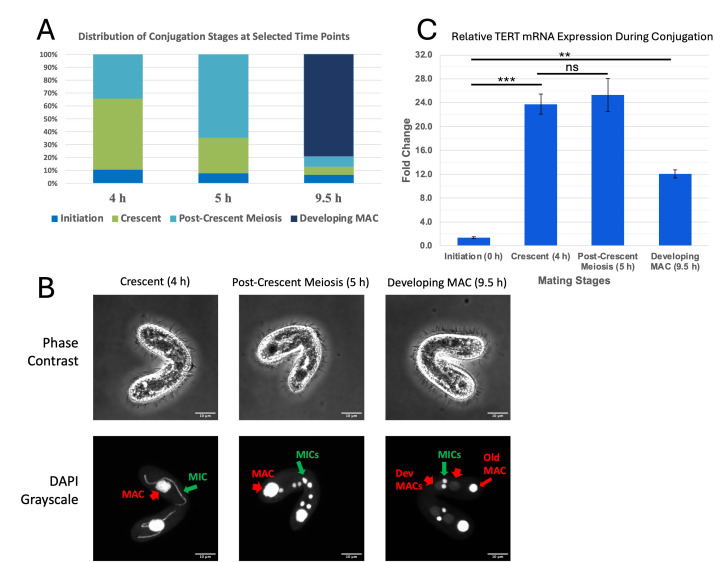



## Description

Telomerase is a ribonucleoprotein enzyme that maintains chromosome ends by synthesizing telomeric DNA repeats, thereby preserving genome stability (Chan & Blackburn, 2004). In ciliates, telomerase adds TTGGGG repeats to chromosome termini and consists of the telomerase reverse transcriptase (TERT) and an RNA template (TER) (Greider & Blackburn, 1987, 1989). Although telomerase regulation is particularly important during sexual development of some higher eukaryotes (Kosebent et al., 2018; MacNeil et al., 2016), its role is especially prominent during the massive genome rearrangements of ciliates such as *Tetrahymena thermophila* when dramatic chromosome restructuring occurs during conjugation (Conover & Brunk, 1986; Yao et al., 2014).

Conjugation in *T. thermophila* proceeds through defined cytological stages involving the micronucleus (MIC) and macronucleus (MAC). During meiotic prophase (crescent stage), the micronucleus elongates 50-fold into a thread-like structure called the crescent stage (Loidl, 2021). This is followed in the post-crescent meiosis by two meiotic divisions that produce four haploid MICs, after which pronuclear exchange and reciprocal fertilization occur. The zygotic nucleus then undergoes mitotic divisions to give rise to developing macronuclei (anlagen), while the parental MAC is degraded. During macronuclear development, chromosomes are fragmented and new telomeres are added de novo to thousands of newly generated chromosome ends (Hamilton et al., 2016; Jacob et al., 2004; Lin et al., 2016; Yao et al., 2014; Zhou et al., 2022).

A previous study identified the TERT gene in *T. thermophila* and reported increased gene expression during conjugation (Bryan et al., 1998). However, these findings relied on end-point PCR and broad sampling intervals, providing only semi-quantitative estimates of transcript abundance. To overcome these limitations, we used reverse transcription quantitative PCR (RT–qPCR), which enables real-time measurement of amplification and more accurate transcript quantification, as well as fluorescence microscopy to identify specific stages of mating. Both the RT–qPCR experiments and cytological analyses were performed using biological and technical replicates at finer time points.

Cells were mated and sampled at multiple, defined time points after mixing. Although conjugation in *T. thermophila* is relatively synchronous (Loidl, 2021), mating populations still contain heterogeneous developmental stages. Since single cells could not be readily excluded, only time points with approximately 65% paired cells were selected for analysis, and only cells visibly in mating pairs were quantified ( Fig. 1A ). Epifluorescence microscopy was used to identify the time points for the selected mating stages, and the percentage of cells at a given hour in a particular stage was calculated. From those calculations, 4 h, 5 h, and 9.5 h after mixing were when most cells were in the crescent stage, the post-crescent meiosis stage, and the macronuclear development stage, respectively ( Fig. 1A ). Representative confocal images of the dominant morphologies at each time point are shown (Fig 1B).

We next examined TERT mRNA expression at 0 h (upon cell mixing), 4 h, 5 h, and 9.5 h during conjugation ( Fig. 1C ). RNA was isolated from mating cells, and expression was compared with that of starved cells, which served as the reference state (1-fold). Primers were designed using an intron-spanning strategy, and expression values were normalized using the candidate reference genes Actin and GAPDH. All conjugation stages exhibited elevated TERT expression relative to starved cells. Specifically, TERT expression peaked during the crescent and post-crescent meiosis stages, reaching more than 25-fold above starved-cell levels, and remained elevated during macronuclear development (~12-fold).

These findings are fairly consistent with those of Bryan et al. (1998), who reported an approximately 45-fold increase in TERT mRNA at 5 hours after strain mixing, followed by a marked decline by 8 hours post-mixing. However, RT–qPCR measures transcript abundance during the exponential phase of amplification, providing more accurate quantification than end-point PCR. In the current study, normalization to two reference genes rather than one, together with fluorescence microscopy, allowed TERT expression to be resolved with greater precision and temporal resolution. The timing of peak TERT mRNA accumulation at 4–5 h post-mixing is notable because extensive de novo telomere addition to fragmented, amplified chromosomes occurs much later during macronuclear development (Yao et al., 2014). One possibility is that early elevated TERT expression ensures sufficient telomerase availability for later chromosomal remodeling during macronuclear development. Alternatively, elevated TERT expression during meiotic prophase may reflect functions associated with chromosome dynamics during meiosis, a stage characterized by extensive chromosome reorganization (Tian et al., 2020).

There are some limitations to this study. First, population-based sampling cannot fully resolve cell-to-cell variability, because conjugation is not perfectly synchronous. Second, Actin and GAPDH were chosen as candidate reference genes based on previous studies rather than being independently validated across the developmental stages examined here. Identification of optimal reference genes throughout the *Tetrahymena* life cycle, including conjugation, will improve future RT–qPCR analyses. Finally, mRNA levels may not directly correspond to telomerase activity during mating because telomerase function also may be influenced by post-transcriptional regulation, protein assembly, and localization.

Future studies will examine the *T. thermophila* TER, a small noncoding RNA for which no introns have been reported (Collins & Gandhi, 1998). Thus, a strategy different from the intron-spanning approach used here for TERT will be required. We also plan to extend these analyses to the filamentous fungus *Aspergillus nidulans* , which lacks the chromosome fragmentation and de novo telomere addition of *T. thermophila* . Notably, vegetative and sexual telomeres in *A. nidulans* are exceptionally short, approximately 110 bp (Day et al., 2025; Wang et al., 2014), raising important questions about how TERT and TER expression are regulated in the context of unusually short but stable telomeres.

## Methods

**Cell Culture and Selection of Conjugation Time Points** . *Tetrahymena thermophila* strains SB210 mating type VI and SB1969 mating type II were obtained from the Tetrahymena Stock Center (RRID:TSC_SD00703 and RRID:TSC_SD00701). Strains were grown at a 1:5 dilution in Neff medium at 30 °C for 24 h, starved in 10 mM Tris-HCl (pH 7.4) for 22 h at 30 °C, mixed in equal volumes, and incubated at 30 °C. Cells were scored as conjugating when two starved cells remained stably attached at their anterior ends while swimming. Because conjugation is not fully synchronous and single cells could not be readily excluded, only time points with >65% paired cells were used for further analysis. Three biological replicates per time point were collected at 30-min intervals between 3–5 h and 8–12 h. Based on these analyses, 4 h, 5 h, and 9.5 h after mixing were selected as time points enriched for crescent-stage cells, post-crescent meiosis, and developing macronuclei, respectively, and were used for RNA isolation and RT–qPCR analysis.

**Epifluorescence DAPI Staging and Confocal Microscopy.** For quantification of cytological stages based on nuclear morphology, cells were fixed in 4% paraformaldehyde in PBS for 20 min at room temperature, washed twice with PBS, stained with DAPI in Vectashield, and sealed with nail polish. Samples were imaged and stages identified using epifluorescence at 100x on a Zeiss Axiovert 100 microscope with a Zeiss Axiocam 208 color camera under phase contrast and UV illumination. DAPI morphology was used to determine the percentage of roughly 80-125 mating pairs at each time point. Cells were categorized according to MIC and MAC morphology. Initiation-stage cells contained a single rounded MIC similar to that observed in starved cells. Crescent-stage cells contained an elongated thread-like MIC. Post-crescent meiosis cells contained two or four MICs. Developing macronuclear-stage cells contained two post-zygotic MICs and developing MACs. To obtain representative confocal images, a 60x objective on a confocal Olympus FV10i under brightfield and UV illumination was used. Optical sections were assembled in ImageJ, and brightness and contrast were adjusted uniformly.

**Primer design.** Actin and GAPDH primers were designed to span at least 170 bp introns, thus preferentially amplifying cDNA over genomic DNA.

**Table:** 

Gene	Sequence	Efficiency	Amplicon Length
GAPDH (TDH1) TTHERM_00551160	F primer: 5’ -GAAGTACGACTCTGCTCACC- 3' R primer: 5’ -GAAAGCACCAGTGGATTCGC- 3'	100.4%	gDNA: 557 bp mRNA: 169 bp
Actin TTHERM_00317000	F Primer: 5’ -GGAGGTTTGACAACAAGAGCAG- 3' R Primer: 5’ -CGCTATTTTCTGGAGCTCTAGG- 3'	86.4%	gDNA: 310 bp mRNA: 137 bp
TERT1 TTHERM_00112560	F Primer: 5’ -CAACTATAATTGCGAGCGAG- 3' R Primer: 5’ -CCTGCTACCTGTAAATAGCC- 3'	83.2%	gDNA: 670 bp mRNA: 328 bp

**RT–qPCR.** Cells were washed with PBS, and total RNA was isolated using the NEB RNA Isolation Kit according to the manufacturer's protocol. RNA concentration was determined spectrophotometrically using a BioTek Synergy HTX multimode reader. cDNA was synthesized with LunaScript RT SuperMix (NEB) and normalized based on initial RNA input by diluting each cDNA reaction to an RNA-equivalent concentration of 100 ng/µL. For each qPCR reaction, 4 µL of diluted cDNA template was added, corresponding to 400 ng of RNA-equivalent input. Reactions were prepared using iTaq Universal SYBR Green Supermix (Bio-Rad) containing 0.5 µM of each primer and were amplified on a CFX Connect Real-Time PCR Detection System (Bio-Rad). The cycling conditions consisted of an initial denaturation at 95 °C for 2 min, followed by 40 cycles of 95 °C for 15 s, 60 °C for 30 s, and 72 °C for 30 s. Three technical replicates from each of three biological replicates were analyzed for each primer pair. Single no-reverse-transcriptase (NRT) and no-template controls (NTC) were included for each primer pair to confirm the absence of genomic DNA and reagent contamination, respectively, but were not included in the relative expression analysis. Expression values were first normalized using the geometric mean of the relative quantities of the two reference genes, Actin and GAPDH. The normalized values were then expressed relative to starved cells at the time of mixing (0 h), which served as the reference sample and were assigned a value of 1-fold. Relative expression was calculated using the Pfaffl method (Pfaffl, 2001; Bustin et al., 2009). A melt curve confirmed amplicon specificity, with each primer pair producing a single peak. Statistical significance was assessed by one-way ANOVA followed by Tukey's post hoc test.
